# Der Preis des späten Facharztzugangs – Auswirkungen auf die Arzneimittelkosten bei rheumatoider Arthritis

**DOI:** 10.1007/s00393-026-01785-6

**Published:** 2026-02-05

**Authors:** Luisa V. Licker, Nicolas Pardey, Kristine Kreis, Jona T. Stahmeyer, Silke Zinke, Dirk Meyer-Olson, Torsten Witte, Kirsten Hoeper, Jan Zeidler

**Affiliations:** 1 Center for Health Economics Research Hannover (CHERH), Leibniz Universität Hannover, Königsworther Platz 1, 30617 Hannover, Deutschland; 2 Fachbereich Wirtschaftswissenschaften, Hochschule Harz, Wernigerode, Deutschland; 3 Klinik für Rheumatologie und klinische Immunologie, Medizinische Hochschule Hannover, Hannover, Deutschland; 4 Stabsbereich Versorgungsforschung, AOK Niedersachsen, Die Gesundheitskasse, Hannover, Deutschland; 5 Rheumatologische Schwerpunktpraxis Berlin, Berlin, Deutschland; 6 Klinik für Rheumatologie und klinische Immunologie, Knappschaft Kliniken Westfalen, Kamen, Kamen, Deutschland

**Keywords:** Routinedaten, Früher Facharztzugang, Kostenanalyse, DMARD-Therapie, Claims data, Early specialist access, Cost analysis, DMARD therapy

## Abstract

**Hintergrund:**

Durch den fachärztlichen Versorgungsmangel in der Rheumatologie in Deutschland kommt es für betroffene Patienten häufig zu einer verzögerten Diagnosestellung, was eine frühzeitige und effektive Behandlung verhindert.

**Ziel der Arbeit:**

Die Studie untersucht die Auswirkungen von frühem vs. spätem Facharztzugang auf die Arzneimittelverordnungen und -kosten.

**Material und Methoden:**

Kostenanalyse im Rahmen der Deliver-Care-Studie mittels GKV-Routinedaten für die Jahre 2015–2020. Patienten mit gesicherter rheumatoider Arthritis (ICD-10 M05/M06) wurden nach dem Zeitpunkt ihres ersten Facharztkontakts gruppiert: früher Zugang (im Diagnosequartal, Q1) vs. später Zugang (Q2–Q4). Für die beiden Gruppen wurden die Arzneimittelkosten verglichen.

**Ergebnisse:**

Mehr als die Hälfte (57,4 %) der M05- und ca. ein Viertel (24,4 %) der M06-Patienten hatten gar keinen Facharztzugang im ersten Jahr nach Stellung der Verdachtsdiagnose. Von den Patienten mit Facharztkontakt (*n* = 3781 Patienten) hatten 82,7 % einen frühen Facharztzugang. Patienten mit späterem Facharztzugang wiesen höhere Medikationskosten auf (4343 € in Q4 vs. 1763 € in Q1; *p* < 0,0001). Eine Sensitivitätsanalyse zeigte, dass Patienten mit spätem Facharztzugang früher auf hochpreisige Medikamente umgestellt wurden als Patienten mit frühem Zugang.

**Diskussion:**

Ein früher Facharztzugang führt zu einer geringeren Verordnungshäufigkeit von Biologika und niedrigeren Arzneimittelkosten. Die Ergebnisse zeigen, dass eine frühzeitige Diagnose und Therapie langfristig nicht nur die Krankheitslast der Patienten reduziert, sondern auch Kosteneinsparungen bei der Behandlung von Patienten mit rheumatoider Arthritis ermöglicht.

**Zusatzmaterial online:**

Die Online-Version dieses Beitrags (10.1007/s00393-026-01785-6) enthält Tab. S1.

## Abstract (en)

**Background:**

Insufficient access to specialist rheumatology services in Germany results in prolonged waiting times and delayed diagnostic confirmation, which impedes timely and effective initiation of therapy.

**Objectives:**

This study examines the impact of early versus delayed access to specialist care on medication prescribing patterns and associated costs.

**Materials and methods:**

A cost analysis was conducted within the Deliver-Care study using health insurance claims data from 2015–2020. Patients with a confirmed rheumatoid arthritis diagnosis (ICD-10 M05/M06) were stratified by the timing of their initial specialist consultation: early access (within the diagnosis quarter, Q1) versus late access (Q2–Q4). Medication costs were compared across these groups.

**Results:**

More than half (57.4%) of M05 patients and about one quarter (24.4%) of M06 patients had no access to a specialist during the first year after the initial suspected diagnosis. Among patients who did have specialist contact (*n* = 3781), 82.7% obtained early specialist access. Patients with delayed specialist access incurred higher medication costs (€ 4343 in Q4 vs. € 1763 in Q1; *p* < 0.0001). A sensitivity analysis showed that patients with delayed specialist access were switched to high-cost medications earlier than those with early access.

**Conclusion:**

Early specialist access is associated with reduced biologic prescribing and lower medication costs. These findings highlight that timely diagnosis and treatment not only lessen patient disease burden but also generate substantial cost savings in the management of rheumatoid arthritis.

## Hintergrund

Für eine qualitativ hochwertige Versorgung von Patienten mit rheumatoider Arthritis (RA) empfehlen die EULAR und die DGRh das frühzeitige Erkennen von Verdachtsfällen sowie einen frühzeitigen Zugang zu einem internistischen Rheumatologen [3, 19, 21]. Aufgrund des Mangels an Rheumatologen in Deutschland erfolgt der Zugang zur fachärztlichen Versorgung bei den meisten Patienten verzögert – häufig erst mehr als zwölf Wochen nach Symptombeginn [3, 14, 18]. Diese Studie analysiert, welche finanziellen Auswirkungen ein verzögerter Facharztzugang hinsichtlich Arzneimittelverordnung und -kosten hat.

## Einleitung

Ein frühzeitiger Zugang zu einem internistischen Rheumatologen sollte gemäß EULAR-Leitlinien innerhalb von sechs Wochen nach Beginn einer Arthritis erfolgen, um nach Diagnosestellung den sofortigen Beginn einer krankheitsmodifizierenden DMARD-Therapie zu ermöglichen [21]. Der gegenwärtige Mangel an Fachärzten für Rheumatologie stellt eine erhebliche Einschränkung für die Implementierung essenzieller patientenspezifischer Maßnahmen dar, darunter die Festlegung individueller Therapiestrategien, die kontinuierliche Überwachung des Krankheitsverlaufs sowie die Diagnostik und Beurteilung von Begleiterkrankungen [1, 3]. Ein leitliniengerechtes Vorgehen ermöglicht, das Fortschreiten der Erkrankung zu verhindern [2, 15]. Ein zentraler Bestandteil der Versorgung ist die medikamentöse Behandlung. Der Einsatz von DMARDs ist entscheidend für den Erfolg der Therapie. Eine frühzeitig begonnene DMARD-Therapie zeigt Langzeiteffekte bei der Krankheitsaktivität über einen Zeitraum von fünf Jahren und erhöht die Wahrscheinlichkeit, eine Remission zu erreichen [6, 10]. Zudem kann durch eine frühzeitige und zielorientierte Behandlung gemäß einer leitliniengerechten Versorgung die Notwendigkeit hochpreisiger Therapieoptionen wie biologischer (bDMARDs) und zielgerichteter synthetischer Arzneimittel (tsDMARDs) verringert werden [11, 21, 23, 25]. Dies verbessert nicht nur die Lebensqualität und soziale Teilhabe der Betroffenen, sondern reduziert auch die damit verbundenen gesundheitsökonomischen Kosten [5]. Ein relevanter Kostentreiber ist dabei die medikamentöse Behandlung, welche einerseits die Betroffenen, andererseits aber auch das Gesundheits- und Sozialsystem durch höhere Ressourcenverbräuche belastet [21, 24, 26]. Im Rahmen einer Metaanalyse aus 72 Studien konnte ermittelt werden, dass die Arzneimittelkosten bis zu 87 % der direkten Kosten ausmachen [9]. Trotz erwiesener klinischer Vorteile eines frühzeitigen Facharztzugangs bei entzündlich-rheumatischen Erkrankungen fehlen nach unserer Kenntnis bislang in Deutschland Daten zu den wirtschaftlichen Folgen verzögerter fachärztlicher Versorgung. Die vorliegende Studie untersucht daher die Auswirkungen eines verspäteten Facharztzugangs auf Arzneimittelverordnungen und Therapiekosten.

## Methodik

### Datengrundlage

Die vorliegende Kostenanalyse wurde im Rahmen der DELIVER-CARE-Studie (Förderkennzeichen: 01NVF18014) durchgeführt und basiert auf GKV-Routinedaten der AOK Niedersachsen (AOKN). Die Datenbasis der Studie umfasst die Stammdaten der Versicherten sowie abrechnungsrelevante Informationen aus den Leistungssektoren der ambulanten und stationären Versorgung, Arzneimittel sowie Heilmittel. Der Datenzeitraum erstreckt sich von 2013 bis 2020, wobei die Jahre 2015 bis 2020 als Studienzeitraum und die Jahre 2013 und 2014 als Vorbeobachtungszeitraum definiert wurden.

### Studiendesign und Patienten

Innerhalb des Studienzeitraums wurden für die Gesamtstudie alle Versicherten identifiziert, die mindestens eine gesicherte ambulante und/oder stationäre entzündlich-rheumatische Diagnose aufwiesen, zu Beginn des Studienzeitraums volljährig und in den Jahren 2013 bis 2020 durchgängig versichert waren oder im Studienzeitraum verstorben sind. Zur Diagnosevalidierung zur Prüfung des Facharztzugangs waren zwei gesicherte ambulante ICD-Diagnosen M05 und/oder M06 innerhalb eines Jahres oder eine stationäre Diagnose erforderlich (M2Q-Kriterium). Für jeden Versicherten wurde ein individueller Beobachtungszeitraum von acht Quartalen ab erstmaliger Diagnose der entzündlich-rheumatischen Erkrankung definiert^1^. Der Beobachtungszeitraum endete am letzten Tag des Quartals vor dem Versterben oder mit dem Ende der Datenverfügbarkeit am 31.12.2020. Anschließend wurden die Patienten in Gruppen mit frühem und spätem Facharztzugang unterteilt. Ein früher Facharztzugang wurde definiert als ein Facharztkontakt innerhalb des Quartals des Diagnoseindex (Q1), analog zu den EULAR-Empfehlungen [3]. Ein später Facharztzugang lag vor, wenn der Erstkontakt im zweiten, dritten oder vierten Quartal nach der Diagnosestellung erfolgte. Um die verschiedenen Arzneimittelwirkstoffe gemäß ihrer Therapiekosten zu differenzieren, wurden diese in die Kategorien „preiswert“ und „hochpreisig“ eingeteilt. Die Kategorie „preiswert“ enthält dabei die konventionellen synthetischen DMARDs wie Methotrexat und Hydroxychloroquin. Unter der Kategorie „hochpreisig“ subsummieren sich alle abgerechneten biologischen (bDMARDs, z. B. Adalimumab, Tocilizumab) und zielgerichteten synthetischen (tsDMARDs, z. B. Baricitinib, Tofacitinib) DMARDs. Eine vollständige Liste findet sich in Tabelle S1 online.

### Statistische Auswertung

Neben deskriptiven statistischen Methoden wurde die Normalverteilung der Gruppen mithilfe des Shapiro-Wilk-Tests überprüft. Da die untersuchten Merkmale keiner Normalverteilung folgten, wurde der Mann-Whitney-U-Test als nichtparametrisches Verfahren angewendet; *p*-Werte $\leq$ 0,05, wurden als statistisch signifikant betrachtet. Die Datenauswertung wurde mit den statistischen Analyseprogrammen IBM SPSS Statistics (für den Datenimport) sowie SAS 9.4 in Kombination mit dem SAS Enterprise Guide 7.1 (für die Datenaufbereitung und -analyse) durchgeführt.

## Ergebnisse

### Beschreibung der Studienpopulation

**Abb. 1:**
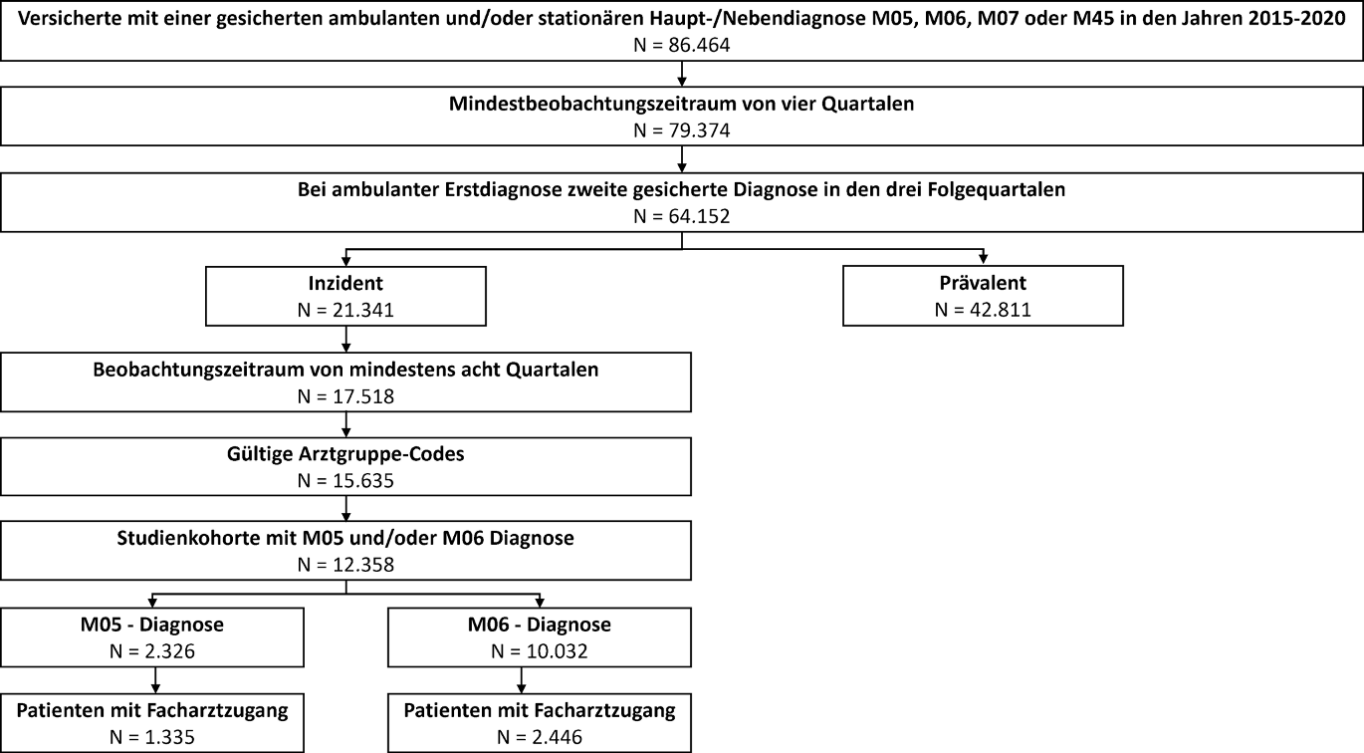
Aufgriff der Studienpopulation

Auf Grundlage des extrahierten Versichertenkollektivs wurden 86.464 Versicherte identifiziert, bei denen zwischen 2015 und 2020 mindestens eine gesicherte ambulante oder stationäre ICD-Haupt- bzw. Nebendiagnose einer entzündlich-rheumatischen Erkrankung dokumentiert wurde. Nach dem Kriterium des Mindestbeobachtungszeitraums von vier Quartalen sowie der Anwendung des M2Q-Kriteriums zur Diagnosevalidierung verblieben 64.152 Versicherte im Kollektiv. Davon entsprach ein Drittel der Definition inzidenter Patienten, d. h. Patienten, bei denen die Diagnose erstmalig im Beobachtungszeitraum gestellt wurde und im Vorbeobachtungszeitraum keine ärztliche Diagnose vergeben wurde. In der Subgruppe der inzidenten Patienten wurde das Kollektiv weiter reduziert. Dazu wurden Versicherte ausgeschlossen, die den für die Fragestellung relevanten Mindestbeobachtungszeitraum von acht Quartalen nicht erfüllten oder keine ausreichende Dokumentation der Arztgruppencodes vorlag. Zudem wurde das Kollektiv auf Patienten mit rheumatoider Arthritis (ICD M05- und M06-Diagnose) eingegrenzt. Von diesen Personen hatten 57,4 % der M05- und 24,4 % der M06-Patienten keinen Facharztzugang im ersten Jahr nach Diagnose, weshalb diese ausgeschlossen wurden. Das finale Analysekollektiv umfasst 3781 Versicherte, von denen 35,3 % der Patienten eine M05- und 64,7 % eine M06-Diagnose aufweisen (Abb. 1).

Die Analysekohorte bestand mit 64,3 % aus Frauen mit einem durchschnittlichen Alter von 61,7 Jahren (Tab. 1).

**Tab. 1:** Patientencharakteristika und Dauer bis zum ersten Facharztkontakt ab Diagnoseindex

		M05 (*n* = 1335)	M06 (*n* = 2446)	M05/M06 (*n* = 3781)
Geschlecht: weiblich *n* (%)		858 (64,3)	1531 (62,6)	2389 (63,2)
Alter Mittelwert (SD)		61,1 (13,8)	62,1 (14,8)	61,7 (14,5)
Dauer bis zum Facharztkontakt *n* (%)	Q1	1055 (79,0)	2073 (84,8)	3128 (82,7)
	Q2	172 (12,9)	210 (8,6)	382 (10,1)
	Q3	76 (5,7)	94 (3,8)	170 (4,5)
	Q4	32 (2,4)	69 (2,8)	101 (2,7)
Komorbiditäten *n* (%)				
F32	Depression	218 (16,3)	410 (16,8)	628 (16,6)
F33	Rezidivierende depressive Störung	79 (5,9)	137 (5,6)	216 (5,7)
E10	Diabetes mellitus Typ 1	31 (2,3)	74 (3,0)	105 (2,8)
E11	Diabetes mellitus Typ 2	208 (15,6)	473 (19,3)	681 (18,0)
E14	Nicht näher bezeichneter Diabetes mellitus	99 (7,4)	223 (9,1)	322 (8,5)
I10	Bluthochdruck/essenzielle Hypertonie	686 (51,4)	1416 (57,9)	2102 (55,6)
I25	Koronare Herzkrankheit (KHK)	143 (10,7)	364 (14,9)	507 (13,4)
E78	Hypercholesterinämie/Hyperlipidämie	360 (26,9)	811 (33,2)	1171 (31,0)

### Zeitraum bis zum ersten Facharztkontakt

In der untersuchten Kohorte, die alle Patienten mit mindestens einem Facharztkontakt im ersten Jahr nach der Diagnosestellung umfasste, wurde die Dauer von der Diagnose bis zum ersten Facharztkontakt auf Quartalsbasis analysiert. Dabei zeigte sich, dass von den Personen mit Facharztzugang 82,7 % der Versicherten diesen bereits im Quartal der Erstdiagnose durch den Zuweiser stattfand. Von der gesamten Studienkohorte entspricht dies lediglich 8,5 %. In den folgenden Quartalen nahm der Anteil deutlich ab: 10,1 % in Q2, 4,5 % in Q3 und 2,7 % in Q4. Zwischen den Diagnosegruppen M05 und M06 sind keine relevanten Unterschiede in der zeitlichen Verteilung des ersten Facharztkontakts sowie bei Komorbiditäten zu beobachten.

In Tab. 2 ist die mittlere Anzahl an Quartalen dargestellt, in denen den Patienten preiswerte und hochpreisige Arzneimittel verordnet wurden. In Abhängigkeit des Facharztzugangs wurden M05-Patienten über den Beobachtungszeitraum im Mittel über eine Dauer von 4,1 bis 4,8 Quartalen preiswerte Arzneimittel verordnet. Hochpreisige Arzneimittel wurden dieser Diagnosegruppe bei frühem Fachartzugang über eine Dauer von 0,4 Quartalen, bei spätem Facharztzugang von bis zu 1,2 Quartalen verordnet. Während 21,9 % der M05-Patienten mit spätem Facharztkontakt (Q4) innerhalb von zwei Jahren bDMARDs und/oder tsDMARDs erhielten, lag dieser Anteil bei Patienten mit frühem Facharztkontakt bei 9,6 %. Bei der M06-Diagnosegruppe wurden über den Beobachtungszeitraum im Mittel über eine Dauer von 2,9 bis 3,7 Quartale preiswerte Arzneimittel verordnet, während hochpreisige Arzneimittel über einen Zeitraum von 0,2 bis 0,6 Quartalen verordnet wurden. Die Ergebnisse einer Sensitivitätsanalyse zeigen, dass Patienten mit erstem Facharztkontakt in Q1 und Q2 im Median nach drei Quartalen hochpreisige Medikamente erhielten, während Patienten mit erstem Facharztkontakt in Q3 bereits nach zwei Quartalen und in Q4 sogar im Median nach nur einem Quartal hochpreisige Medikamente verordnet bekamen. Dies deutet darauf hin, dass ein späterer Facharztkontakt mit einer schnelleren Verordnung hochpreisiger Medikamente assoziiert ist.

**Tab. 2:** Mittlere Anzahl an Quartalen im Follow-up-Zeitraum, in denen den Patienten „preiswerte“ und „hochpreisige“ Arzneimittel verordnet wurden in Abhängigkeit vom Zeitpunkt des Facharztzugangs

	Medikationsgruppen		Quartal des Facharztzugangs nach Diagnoseindex			
			Q1	Q2	Q3	Q4
*M05/M06*	Preiswert	Keine Medikation	31,5 %	23,30 %	20,6 %	27,7 %
		Mittelwert (SD)	3,4 (2,9)	3,8 (3,0)	4,2 (2,9)	3,6 (3,1)
		Median	3	4	4	3
	Hochpreisig	Keine Medikation	93,3 %	90,3 %	84,7 %	87,1 %
		Mittelwert (SD)	0,3 (1,1)	0,3 (1,1)	0,6 (1,7)	0,7 (1,9)
		Median	0	0	0	0
*M05*	Preiswert	Keine Medikation	17,8 %	13,9 %	15,8 %	15,6 %
		Mittelwert (SD)	4,3 (2,8)	4,7 (2,9)	4,8 (2,8)	4,1 (2,8)
		Median	5	5	5	4
	Hochpreisig	Keine Medikation	90,4 %	86,6 %	81,6 %	78,1 %
		Mittelwert (SD)	0,4 (1,2)	0,4 (1,2)	0,7 (1,6)	1,2 (2,5)
		Median	0	0	0	0
*M06*	Preiswert	Keine Medikation	38,5 %	30,9 %	24,5 %	33,3 %
		Mittelwert (SD)	2,9 (2,9)	3,2 (2,9)	3,7 (2,9)	3,4 (3,2)
		Median	2	3	4	3
	Hochpreisig	Keine Medikation	94,7 %	93,3 %	87,2 %	91,3 %
		Mittelwert (SD)	0,2 (1,0)	0,2 (1,0)	0,6 (1,7)	0,4 (1,5)
		Median	0	0	0	0

Als *preiswert* werden csDMARDs, als *hochpreisig* bDMARDs und tsDMARDs bezeichnet

### Kosten der Medikation

**Abb. 2:**
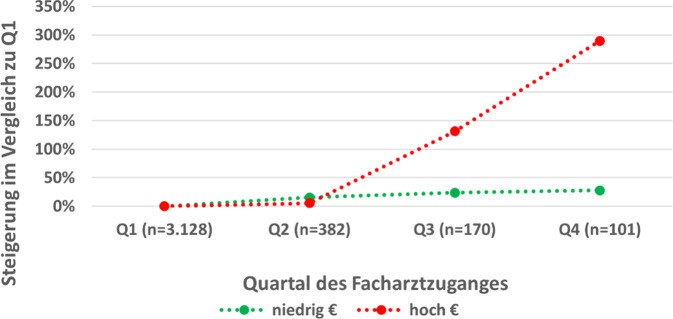
Kostenentwicklung nach Quartal des Facharztzugangs bei RA

Die Analyse der Medikationskosten wies signifikant höhere durchschnittliche Arzneimittelkosten im Beobachtungszeitraum von mindestens acht Quartalen bei Patienten mit späterem Facharztkontakt auf (*p* < 0,0001). Mit jedem Quartal der Verzögerung des ersten Facharztkontakts war eine Steigerung der durchschnittlichen Kosten zu beobachten (Q1: 1763 €; Q2: 2207 €, Q3: 3705 €; Q4:4343 €). Die Kosten der medikamentösen Therapie verdoppeln sich bei einem Zugang im Q3 im Vergleich zu Q1. Bei einem Zugang in Q4 beträgt die durchschnittliche Steigung 178 % (Abb. 2).

### Getrennte Analyse nach Diagnosen

Bei der getrennten Betrachtung nach Diagnosegruppen zeigten sich ebenfalls signifikante Unterschiede. Patienten mit der Diagnose M05 und frühem Facharztzugang hatten im Beobachtungszeitraum durchschnittliche Arzneimittelkosten von 2367 €, während die Kosten bei späterem Zugang auf 3582 € anstiegen (*p* = 0,0062). In der Diagnosegruppe M06 liegen die durchschnittlichen Arzneimittelkosten bei frühem Facharztzugang in Q1 bei 1455 €, während sie sich bei spätem Zugang auf 2252 € belaufen (*p* = 0,001).

## Diskussion

Bei chronischen Erkrankungen, wie der RA, ist eine frühzeitige und intensive Behandlungen essenziell, um eine Krankheitsremission zu erreichen [4, 21, 22]. In unserer Studienkohorte hatten mehr als die Hälfte (57,4 %) der M05- und ca. ein Viertel (24,4 %) der M06-Patienten innerhalb des ersten Jahres keinen Facharztkontakt. Die klinischen Auswirkungen der Verzögerung sind in der Literatur bereits ausführlich beschrieben [15, 16]. Die ökonomischen Folgen eines verspäteten Zugangs zu Fachärzten hinsichtlich medikamentöser Therapie sind jedoch unzureichend untersucht, weshalb die vorliegende Studie dort ansetzt. Anhand von GKV-Routinedaten können die Auswirkungen eines späten Facharztzugangs auf die Verschreibung von preiswerten und hochpreisigen Arzneimitteln für den gesamten ambulanten Versorgungsbereich abgebildet werden. csDMARDs verursachen vergleichsweise geringe Jahreskosten, die je nach Wirkstoff und Darreichungsform zwischen etwa 100 € und 930 € liegen [12]. Deutlich höher sind die Kosten für bDMARDs, die – abhängig von Wirkstoff, Applikationsform und Generikaverfügbarkeit – zwischen ca. 11.000 € und 22.000 € pro Jahr betragen. Für tsDMARDs liegen die jährlichen Therapiekosten im Bereich von etwa 15.000 € bis 16.000 €. Bei bDMARDs und tsDMARDs ist häufig eine Kombinationstherapie mit Methotrexat erforderlich, was die Gesamtkosten weiter erhöht [12]. Wir konnten zeigen, dass Patienten, die erst spät einen Facharzt aufsuchten, im Vergleich zu einem frühen Facharztkontakt häufiger und früher hochpreisige Arzneimittel verordnet werden. 82,7 % der Patienten hatten im Quartal der Erstdiagnose einen Facharztkontakt, wobei sich zeigte, dass Patienten mit verzögertem Zugang häufiger eine intensivere und kostenintensivere Therapie benötigen. Die Sensitivitätsanalysen unterstützen diesen Befund: Patienten mit spätem Facharztkontakt erhielten schneller eine Verordnung hochpreisiger Medikamente als jene mit frühem Facharztkontakt. Die Kosten für Patienten ohne einen Facharztzugang konnten wir nicht messen, weshalb eine langfristige Untersuchung notwendig ist. Wir können jedoch mit unseren Ergebnissen vermuten, dass die Arzneimittelkosten dieser Kohorte noch hochpreisiger ausfallen dürften, da ein besonders relevanter Aspekt die steigende Kostenbelastung mit zunehmender Verzögerung des Facharztkontakts ist. Bei Patienten mit spätem Facharztzugang stiegen die durchschnittlichen Kosten für Medikamente auf mehr als das Doppelte an, im Vergleich zu Patienten mit frühem Facharztkontakt. Mulligen et al. (2024; [17]) untersuchten erstmals, wie sich eine frühe Diagnosestellung (< 12 Wochen nach Symptombeginn) und ein frühzeitiger Behandlungsbeginn auf die Behandlungskosten auswirken. In einer Kohorte von 431 RA-Patienten der Leiden Early Arthritis Clinic (2011–2017) verglichen sie über fünf Jahre die Kosten der DMARD-Verordnungsdaten. Es wurde festgestellt, dass die Behandlungskosten bei seronegativer RA bei einem späten Zugang um 316 % höher waren. Bei seropositiver RA stiegen die Kosten um 46 % an, wenn die Diagnose länger als ein Jahr nach Symptombeginn gestellt wurde und Biologika verwendet wurden. Lorenz et al. (2019; [15]) weisen in ihrer Übersichtsarbeit darauf hin, dass nach der aktuellen Studienlage eine frühzeitige Vorstellung beim Facharzt die Therapierbarkeit entzündlich-rheumatischer Erkrankungen erheblich verbessert und dadurch den Einsatz kostenintensiver Therapeutika, wie Biologika, deutlich verringern kann. Eine frühzeitige Versorgung ist daher nicht nur aus individueller und ethischer Sicht, sondern auch unter versorgungsstrategischen und gesundheitsökonomischen Gesichtspunkten von großer Bedeutung [15, 17, 20]. Trotz der aufschlussreichen Erkenntnisse im Zusammenhang zwischen Facharztzugang und Medikation unterliegt unsere Studie mehreren Limitationen. Die Nutzung von bundeslandspezifischen Daten der AOK schränkt die Generalisierbarkeit der Ergebnisse auf andere Regionen ein. Zudem fehlt eine Langzeitanalyse ökonomischer Effekte, wodurch die tatsächlichen Kostenimplikationen verzögerter Therapien möglicherweise unterschätzt werden. Der Anteil der M06-Diagnosen im Vergleich zu den M05-Diagnosen ist höher als in anderen Kohorten. Angaben über klinische Charakteristika; wie z. B. Krankheitsaktivität; konnten aufgrund der Datenbasis nicht herangezogen werden. Zudem wurden Patienten ohne Zugang zu fachärztlicher Versorgung ausgeschlossen. Diese Gruppe könnte sich hinsichtlich soziodemografischer Merkmale systematisch unterscheiden und ist zugleich relevant für die Abbildung von Versorgungsdefiziten. Vor diesem Hintergrund könnten die vorliegenden Ergebnisse den tatsächlichen Effekt unterschätzen. Die letztlich für die Auswertung verfügbare Kohorte umfasste 3781 Personen. Lediglich 10,1 % der Studienkohorte hatte einen späten Facharztzugang. Dieses wird mit der frühen Gruppe verglichen, welche einen deutlich höheren Anteil der Studienkohorte ausmacht. Durch diese Ungleichheit kann es zu einer Verzerrung der Ergebnisse kommen. Die Definition von frühem und spätem Kontakt basiert auf der leitliniengerechten Versorgung. Nichtsdestotrotz könnte diese, gerade an den Quartalsgrenzen, als recht streng erachtet werden. Die Ergebnisdarstellung in Quartalen soll dem entgegenwirken und für Transparenz sorgen. Ein weiterer methodischer Aspekt betrifft die Gruppierung der Medikamente in preiswerte und hochpreisige Medikation. Diese Einteilung basiert auf dem Spektrum der gängigen Medikation zum Zeitpunkt der Studiendurchführung sowie auf der Validierung anhand medizinischer Expertise. Nichtsdestotrotz unterstreichen die Ergebnisse dieser Studie die wirtschaftlichen Vorteile einer frühen Intervention, da frühe Behandlungsstrategien nicht nur das Fortschreiten der Krankheit verlangsamen, sondern auch die Notwendigkeit einer Verordnung hochpreisiger Biologika reduzieren können. Weitere Untersuchungen sollten sich darauf konzentrieren, welche Faktoren zu späteren oder keinen Facharztkontakten führen und wie diese durch gezielte Interventionsstrategien optimiert werden können. Erste Lösungsansätze bietet bspw. die Integration rheumatologischer Fachassistenz in die Versorgung [7, 8, 13].

## Fazit für die Praxis

- Ein früher Facharztkontakt innerhalb kurzer Zeit ist von hoher Bedeutung und könnte zu einer kosteneffizienten sowie optimalen Patientenversorgung beitragen.
- Die Ergebnisse unserer Studie deuten darauf hin, dass Patienten mit einem verzögerten Facharztzugang in den folgenden zwei Jahren höhere durchschnittliche Medikamentenkosten aufweisen. Darüber hinaus ist ein Zusammenhang zwischen hochpreisiger Medikation und einer Verzögerung des ersten Facharztkontakts erkennbar.
- Diese Ergebnisse zeigen die Notwendigkeit, den Zugang zur rheumatologischen Fachversorgung zu verbessern.
- Ein frühzeitiger Zugang könnte neben der Therapieoptimierung auch Kostenersparnisse für Arzneimittel bewirken. Aufgrund dessen gewährleistet ein frühzeitiger Zugang zu einer qualifizierten rheumatologischen Fachversorgung eine kosteneffiziente und optimale Patientenversorgung.

## Supplementary Information

Tab. S1: ATC-Codes zur Identifikation preiswerter und hochpreisiger Medikation

## Data Availability

Die GKV-Routinedaten, die dieser Publikation zugrunde liegen, sind aufgrund von Datenschutzbestimmungen und gesetzlichen Vorgaben nicht öffentlich verfügbar. Der vollständige Code zur Datenaufbereitung und statistischen Analyse kann auf begründete Anfrage beim korrespondierenden Autor angefordert werden.

## Footnotes

1 Da die Diagnosedokumentation in GKV-Routinedaten nur quartalsweise erfolgt, wurde der erste Tag des Quartals als Diagnoseindex definiert.
