# Supplementary material for: Consequences of late access to specialist care—effect on medication costs in rheumatoid arthritis
Source: Z Rheumatol. 2026 Feb 5;85(4):272–9. [Article in German] doi: 10.1007/s00393-026-01785-6 (PMC13124890; doi:10.1007/s00393-026-01785-6)
Supplement: Supplementary file 1 — Tab. S1: ATC-Codes zur Identifikation preiswerter und hochpreisiger Medikation [file 393_2026_1785_MOESM1_ESM.pdf]

Supplementary Information

Tabelle S1: ATC-Codes zur Identifikation preiswerter und hochpreisiger Medikation

**Preiswerte Medikation**		**Hochpreisige Medikation**			
ATC-Code	Wirkstoff	ATC-Code	Wirkstoff	ATC-Code	Wirkstoff
**csDMARDs**		**bDMARDs/Biologika**		**tsDMARDs**	
L01BA01	MTX	L04AA24	Abatacept	L04AA37	Baricitinib
L04AX03	MTX	L04AB04	Adalimumab	L04AA29	Tofacitinib
M01CX01	MTX	L04AC03	Anakinra	L04AA44	Upadacitinib
L04AA13	Leflunomid	L04AB05	Certolizumab	L04AA45	Filgotinib
A07EC01	Sulfasalazin	L04AB01	Etanercept	L04AA32	Apremilast
M01CX02	Sulfasalazin	L04AB06	Golimumab		
P01BA01	Chloroquin	L04AB02	Infliximab		
P01BA02	Hydroxychloroquin	L01XC02	Rituximab		
L04AX01	Azathioprin	L04AC07	Tocilizumab		
		L04AC14	Sarilumab		
		L04AC05	Ustekinumab		
		L04AC16	Guselkumab		
		L04AC10	Secukinumab		
		L04AC13	Ixekizumab		
		L04AC21	Bimekizumab		

Übersicht der zur Identifikation der Medikation verwendeten ATC-Codes, basierend auf der Klassifikation in preiswerte und hochpreisige Medikation. Die Einteilung erfolgte nach csDMARDs, bDMARDs/Biologika und tsDMARDs, wobei die Auswahl medizinisch validiert wurde.
